# Evolution of the Pax-Six-Eya-Dach network: the calcisponge case study

**DOI:** 10.1186/2041-9139-5-23

**Published:** 2014-06-23

**Authors:** Sofia AV Fortunato, Sven Leininger, Maja Adamska

**Affiliations:** 1 Sars International Centre for Marine Molecular Biology, University of Bergen, Thormøhlensgt. 55, Bergen 5008, Norway; 2 Department of Biology, University of Bergen, Thormøhlensgt. 55, Bergen 5008, Norway; 3 Current address: Institute of Marine Research, Nordnesgaten 50, Bergen 5005, Norway

**Keywords:** Calcisponges, *Sycon*, *Eyes absent*, *Pax*, *Six*, Sensory cells

## Abstract

**Background:**

The *Pax-Six-Eya-Dach* network (PSEDN) is involved in a variety of developmental processes, including well documented roles in determination of sensory organs and morphogenesis in bilaterian animals. Expression of PSEDN components in cnidarians is consistent with function in sensory organ development. Recent work in demosponges demonstrated the presence of single homologs of *Pax* and *Six* genes, and their possible involvement in morphogenesis, but the absence of the remaining network components. Calcisponges are evolutionarily distant from demosponges, and the developmental toolkits of these two lineages differ significantly. We used an emerging model system, *Sycon ciliatum*, to identify components of the PSEDN and study their expression during embryonic and postembryonic development.

**Results:**

We identified two *Pax*, three *Six* and one *Eya* genes in calcisponges, a situation strikingly different than in the previously studied demosponges. One of the calcisponge *Pax* genes can be identified as *PaxB*, while the second *Pax* gene has no clear affiliation. The three calcisponge *Six* genes could not be confidently classified within any known family of *Six* genes. Expression analysis in adult *S. ciliatum* demonstrated that representatives of *Pax*, *Six* and *Eya* are expressed in patterns consistent with roles in morphogenesis of the choanocyte chambers. Distinct paralogues of *Pax* and *Six* genes were expressed early in the development of the putative larval sensory cells, the cruciform cells. While lack of known photo pigments in calcisponge genomes precludes formal assignment of function to the cruciform cells, we also show that they express additional eumetazoan genes involved in specification of sensory and neuronal cells: *Elav* and *Msi*.

**Conclusions:**

Our results indicate that the role of a *Pax-Six-Eya* network in morphogenesis likely predates the animal divergence. In addition, *Pax* and *Six*, as well as *Elav* and *Msi* are expressed during differentiation of cruciform cells, which are good candidates for being sensory cells of the calcaronean sponge larvae.

## Background

In insect and vertebrate model systems, *Pax*, *Six*, *Eyes absent* (*Eya*) and *Dachshund* (*Dach*) form a network (PSEDN) interconnected by a series of protein-protein and protein-DNA interactions [1,2]. This network is often referred to as Retinal Determination Gene Network (RDGN), although it is involved in a variety of developmental processes in addition to eye development, including roles in morphogenesis of other sensory organs, kidneys and the branchial arches [3-5]. It has been suggested that the insect and vertebrate PSEDN/RDGN are not homologous [6], although studies in cnidarians indicate deep evolutionary roots of this network. In particular, it has been demonstrated that members of the Pax, Six and Eya families are expressed during neural development and sensory organ formation in a wide range of cnidarians [7-15]. In demosponges, two components of the PSEDN have been identified, *PaxB* and *Six1/2*[16,17]. A recent study in the freshwater demosponge *Ephydatia muelleri* shows that these genes are co-expressed, potentially interact and are likely involved in juvenile/adult morphogenesis [16,17].

While sponges lack a nervous system, larvae of some species have well defined sensory cells, organized into simple organ-like structures [18,19]. For example, the parenchymella-type larvae of *Amphimedon queenslandica* have a pigmented ring equipped with long “steering” cilia at their posterior pole known as the sensory organ of the larva. Although opsin is not found in the *A. queenslandica* genome, the larval phototactic behavior [20] is likely mediated by cryptochrome [21], which has also been suggested to participate in light reception in adult tissue of another demosponge, *Suberites domuncula*[22]. Significantly, *AmqCry2* expression is associated with the pigment ring [23]. Unfortunately, no information regarding expression of *Pax* or *Six* genes during development of the pigment ring is published, making it impossible to predict whether the ancestral PSEDN function was related to morphogenesis only or both morphogenesis and sensory organ formation.

We have recently began developing *Sycon ciliatum* as a model representing calcisponges (subclass *Calcaronea*), a lineage evolutionarily distant from demosponges and appearing to significantly differ from demosponges in its gene content [24-27]. Embryonic and postembryonic development of syconoid calcaronean species is well studied, allowing us to relate gene expression patterns to developmental events. Importantly, different stages of radial (choanocyte) chamber morphogenesis can be compared in a single specimen fixed during the growth phase: when the asconoid body plan of the juvenile gives rise to the syconoid body plan of the adult, radial chambers form around the original central choanocyte chamber and continue to develop sequentially from bottom to top, with the region just under the osculum remaining in asconoid organization [28]. The amphiblastulae larvae of calcisponges from the subclass Calcaronea are strikingly different from the parenchymellae [25,29,30]. Amphiblastulae are composed of only three cell types of embryonic origin: macromeres, micromeres, and four cruciform cells distributed around the “equator” and conveying unique tetra-radial symmetry to the larva [24,31]. While the macromeres and micromeres participate in formation of the juvenile body upon metamorphosis, the cruciform cells degenerate upon settlement [32]. The function of cruciform cells has not been studied experimentally, but based on ultrastructure examination it has been suggested that they might act as photoreceptors [33]. Intriguingly, differentiating cruciform cells of *S. ciliatum* express *SoxB*[24], a transcription factor involved in bilaterian neurogenesis [34,35] and expressed in cnidarian neurosensory cells [36]. In addition, they express several genes which, while clearly having multitude of roles in animal development, are also implicated in specification of neuronal cell types in eumetazoans: components of the Wnt pathway (*dvl*, *tcf* and *beta-catenin*), *Smad1/5* and *nanos*, lending support to the notion that they could be sensory cells [25,37-39].In this study we chose to address the evolution of the PSEDN by studying expression of potential components of this network in *S. ciliatum*, focusing on the cruciform cells as the likely sensory cells of the larvae, and on the adult morphogenesis represented by formation of the radial (choanocyte) chambers.

We searched the genomic and transcriptomic datasets of *S. ciliatum* and a second calcaronean species, *Leucosolenia complicata,* for genes encoding the components of the PSED network. To gain additional insight into identity of the cruciform cells, we also searched for genes encoding known proteins involved in photoreception (opsin and cryptochrome), and the RNA binding proteins Elav and Musashi, which are involved in specification of neurosensory cells in eumetazoans. In this paper, we report that calciponge genomes contain an ortholog of the *Eya* gene, which has not been previously reported in demosponges. We have not identified opsin and dachshund in calcisponges, which is consistent with the absence of these genes in demosponges. On the other hand, cryptochrome, which is present in demosponges, and likely responsible for light perception in the demosponge larvae, is absent from the calcisponge genomes. Expression of *Pax, Six, Eya, Msi* and *Elav* genes in *Sycon ciliatum* was studied by *in situ* hybridization. Here we show that *Pax, Six* and *Eya* genes are co-expressed during morphogenesis of the radial chambers, and that *Pax* and *Six*, as well as *Elav* and *Msi* are co-expressed during formation of cruciform cells.

## Methods

### Sequence retrieval, alignment and phylogenetic analyses

*Sycon ciliatum* and *Leucosolenia complicata Pax*, *Six* and *Eya* genes were identified by BLAST searches of *Sycon* and *Leucosolenia* draft genomes (a preliminary draft of *Leucosolenia*) and transcriptomes as previously described [24] using specific domains from the following taxa: Bilateria, *Branchiostoma floridae* and *Mus musculus*; Porifera, *Amphimedon queenslandica*; Cnidaria, *Nematostella vectensis*.

For *Eya*, alignments were performed using the conserved ED domain. For *Pax* genes two alignments were performed: in the first alignment, the complete paired domain (PD) was included and in the second alignment the truncated RED-PD domain was used. Lack of homeodomains in the calcisponge *Pax* sequences precludes homeodomain-based phylogenetic analyses. For *Six* genes, the homeodomain along with the extended *sine oculis* domain was used. A combination of ClustalX and MUSCLE was used for the alignments, which were manually corrected where necessary.

The amino acid substitution model of protein evolution was determined by ProtTest 3.0 [40]. For all analyses the best model of protein evolution was LG + G, except for the analysis of the complete PD domain of *Pax* genes where invariant site gamma LG + G + I was optimal. Bayesian and maximum likelihood (ML) analyses were undertaken on conserved regions. For the MrBayes 3.1 analyses [41] (with LG model incorporated by in-house modification), a set of four independent Metropolis-coupled Markov Chain Monte Carlo (MCMC) were sampled every 1,000^th^ generation. Two Bayesian analyses were run for each dataset from 1 to 10 million generations, depending on the dataset. Convergence was assessed by plotting the log likelihood against the number of generations using Tracer v1.4 [42]. The analysis were stopped when the split frequency between the two runs was lower than 0.01. After the removal of an appropriate burn-in (20 to 25% in most cases), the consensus trees were visualized with FigTree v1.4.0 [43]. The ML analysis was performed using PhyMl 3.0 [44] as follows: To provide a starting tree for the bootstrap analysis, two rounds of PhyMl analysis, each starting from five random trees, were run using the following command line: -i align.phy –d aa –f e –m LG –c 4 –a value –v value –rand_start –s NNI. The better of the two resulting ML trees (the tree with better likelihood value) was selected as an input tree for 1,000 bootstrap analysis using the following command line: -i align.phy -d aa -f e -m LG -c 4 -a value -v value -u best_random_tree.nwk -b 1,000 -s NNI.

### Molecular analyses: RT-PCR and *in situ* hybridization

Sample collection, fixation, PCR amplification of genes, sequencing, probe production and *in situ* hybridization in sliced sponges containing different reproductive stages and in small adult *Sycon ciliatum* specimens were performed as described previously [24].

## Results

### Two Pax genes are found in *Sycon* and one in *Leucosolenia*

**Figure 1:**
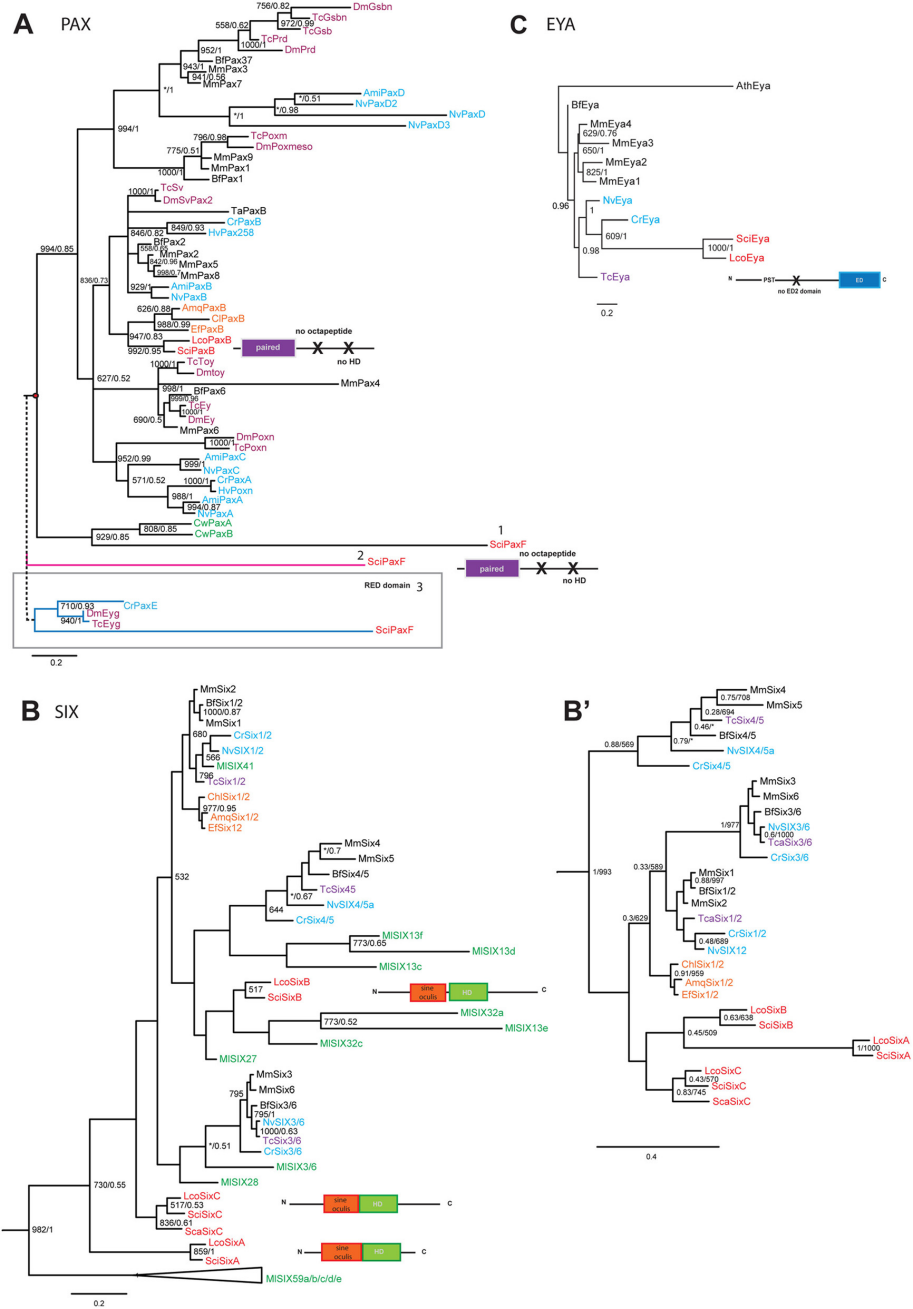
**Phylogenetic analyses of the Six, *Eya *and *Pax *genes. A**, Bayesian phylogenetic tree of Pax genes inferred from the paired domain. The red dots indicate the different positions of *SciPaxF*: 1, position of *SciPaxF* when the complete the PD domain was used to infer the phylogeny; 2, position of *PaxF* when removing ctenophore sequences and 3, when using the RED domain (S6) and including *CrPaxE* and Arthropod *Eyg* genes. **B**, The maximum likelihood tree was inferred from the Six homeodomain. The Six tree was rooted using the TALE class homeodomain as an outgroup. **B**’, Bayesian tree inferred from the Six homeodomain without including *Mnemiopsis* Six genes. **C**, Bayesian tree of Eya ED domain. The EYA tree was rooted using *Arabidopsis thaliana* (At) *Eya-like* gene. ML bootstrap values greater than 500 (left) and posterior probabilities generated by MrBayes greater than 0.5 (right) are displayed. Asterisks indicate the differences in the position of a given gene. Names are prefixed as follows: Porifera: Calcisponges, Sca = *Sycon calcavaris*; Sci = *Sycon ciliatum* and Lco = *Leucosolenia complicata*. Demosponges, Amq *= Amphimedon queenslandica*; Ef = *Ephydatia fluviatilis*; Chl = *Chalinula loosanoffi*. Ctenophora: Ml = *Mnemiopsis leidyi* and Cw = *Coeloplana willeyi*. Cnidaria: Anthozoa, Nv = *Nematostella vectensis and* Hydrozoa Cr *= Cladonema radiatu*; Hv = *Hydra vulgaris*; Ami = *Acropora millepora*. Bilateria: Protostomia, Tc = *Tribolium castaneum*.; Dm = *Drosophila melanogaster*. Deuterostomia: Bf = *Branchiostoma floridae* and Mm = *Mus musculus*.

Two *Pax* genes were found in *Sycon* and one in *Leucosolenia*. As the *Leucosolenia* dataset is less extensive than the *Sycon* one, it is possible that our current analysis can miss a *Leucosolenia* sequence. In contrast to the demosponges’ *PaxB* with a recognizable partial homeodomain and an octapeptide, the *Pax* genes in calcisponges do not appear to contain a homeodomain or octapeptide. Both *Sycon Pax* genes contain an intron in the PD domain (Additional file 1) corresponding to the intron-exon boundaries found in cnidarians [45] and in other sponges [16,46]. The phylogenetic analysis of the PD of Pax (Figure 1C, Additional file 2) shows that both calcisponges have a single ortholog of *PaxB* and confirms the affiliation of demosponges *PaxB* genes as previously reported [16,46]. Several phylogenetic analyses were performed to determine affiliation of the second *Pax* gene in *Sycon* (Figure 1A). The first analysis used an alignment of the complete PD domain (Figure 1A, position 1), and indicated affiliation of this gene with the ctenophore *Coeloplana willeyi Pax* genes [47]. For the second analysis we removed the *C. willeyi* sequences from the alignment, and in this analysis the second *Sycon Pax* gene did not affiliate with any subfamily of *Pax* genes (Figure 1A, position 2). Finally, in the third analysis (Figure 1A, position 3), we used the partial PD domain (RED motif) and included arthropod *eye gone* (*Eyg*) genes and *Cladonema radiatum PaxE* gene. The result of the analysis shows that this second *Pax* gene fell within the PaxE subfamily (the *Eyg* subfamily) but this association was not supported (see also Additional file 3). Due to the unclear affiliation of the second *Pax* gene in *Sycon*, we decided to name it *PaxF*, following the next letter in the classification of *Pax* genes. Importantly, in none of our analyses *SciPaxF* affiliated with Pax1/9 and/or Pax3/7 subfamilies and thus it does not provide additional support for the notion of Pax duplication before divergence of Porifera [48].

### SINE class family is expanded in calcisponges

We found three *Six* genes in each of the *Sycon* and *Leucosolenia* genomes corresponding to the SINE class of homeobox genes. All of them had the characteristic Six homeodomain with lysine at position 50 and the *sine oculis* DNA binding domain situated at the -N terminal to the homeodomain (Additional file 4), as seen in previously classified *Six* genes [49]. Additionally, we found two genes (*SciHD35531*and *LcoHD71216*) that contained partial *sine oculis* domains and homeodomains which displayed similarity to both the TALE and SINE gene classes. The homeodomains of *SciHD35531* had a four amino acid insertion, instead of the three typically observed in TALE homeodomains (Additional file 5).

The first phylogenetic analysis was based on the homeodomain sequences and included the entire expanded complement of ctenophore *Six* genes [50] (Figure 1B). This analysis correctly separated the cnidarian and bilaterian sequences into the three recognized families (Six1/2, Six3/6 and Six4/5) and confirmed the affiliation of demosponge *Six* genes within the Six1/2 family [16,17,46] albeit with low support value (Figure 1B). Among the ctenophore sequences, *MlSix41* nested within the Six1/2 family, consistent with the presence of the ETSY motif in its homeodomain, while *MlSix36* and *MlSix28* grouped with the Six3/6 family as described [50]. Surprisingly, none of the calcisponge sequences associated with demosponge sequences or any other recognized Six families. Because of unclear affiliation, calcisponge *Six* genes are referred to as *SixA*, *SixB* and *SixC* (with the *SixC* sequences being most similar to the *SixC* sequence from *Sycon calcaravis*, *ScaSixC*[47]). In the tree presented on Figure 1B, *Sci/LcoSixB* genes associated with several of the ctenophore sequences, and this grouping receives moderate support in the ML analysis; while *SciSixA* and *SciSixC* fell completely outside of the recognized Six families. Given the difficulty in assigning the ctenophore *Six* genes and the fact that many of them are on long branches, we suspected that affiliations of calcisponge and *Mnemiopsis Six* genes might represent an artefact of long-branch attraction. We have thus carried out additional analyses with limited complements or completely without the ctenophore sequences, utilizing either only the homeodomain (Figure 1B’ and Additional file 6), or the SINE domain together with the homeodomain (Additional file 7). In particular, we were hoping to differentiate between scenarios in which the three calcisponge *Six* genes are all descended from a *Six1/2* ancestral sequence, and are a result of family expansion in the calcisponge lineage, or are remnants of ancestral sequences, which are preserved in ctenophore and calcisponge genomes and lost in all others. In the homeodomain-only analysis without the ctenophore sequences, the calcisponge sequences clustered together, although this grouping did not receive significant support (Figure 1B’). In the tree utilizing the SINE domain, the calcisponge sequences did not form a monophyletic clade, but all of them branched basally (Additional file 7). Significantly, the grouping of the demosponge *Six* genes with the Six1/2 family was sensitive to taxon sampling. However, all demosponge *Six* and the calcisponge *SixB* homeodomains have the characteristic Six1/2 family 'ETSY' motif, and *SixC* sequences have a similar ‘ETNY’ motif ( [50] and Additional file 5). Altogether, the results of the phylogenetic analyses indicate that the calcisponge *Six* genes may represent a specific diversification of the SINE class in calcareous sponges, similar to the one observed in the ctenophores [50], although the exact relationships between the paralogs remain unclear.

### *Eyes absent* (Eya) is present in calcisponges

We found single orthologs of the eyes absent gene, *Eya,* in the two calcisponges*.* The *Eya* gene has not been previously reported in demosponges. In both species, the predicted proteins have a conserved C-terminal amino-acid domain, the *Eya* domain (ED) but not the N-terminal ED2 domain (Additional files 8 and 9). The phylogenetic analysis in Figure 1C shows that the calcisponge *Eya* genes affiliate with the cnidarian *Eya* genes with moderate support values. *Eya* gene containing the ED domain and P/S/T rich region is found in the choanoflagelate *Monosiga brevicollis*[13], indicating that *Eya* was lost in demosponges. The presence of the ED domain and the P/S/T region but not the ED2 region of the *Eya* gene corroborates the notion that the ED2 domain was established in the last common ancestor of bilaterians and cnidarians [13].

### Pax and Six genes, but not Eya are expressed during embryogenesis and in the larvae

**Figure 2:**
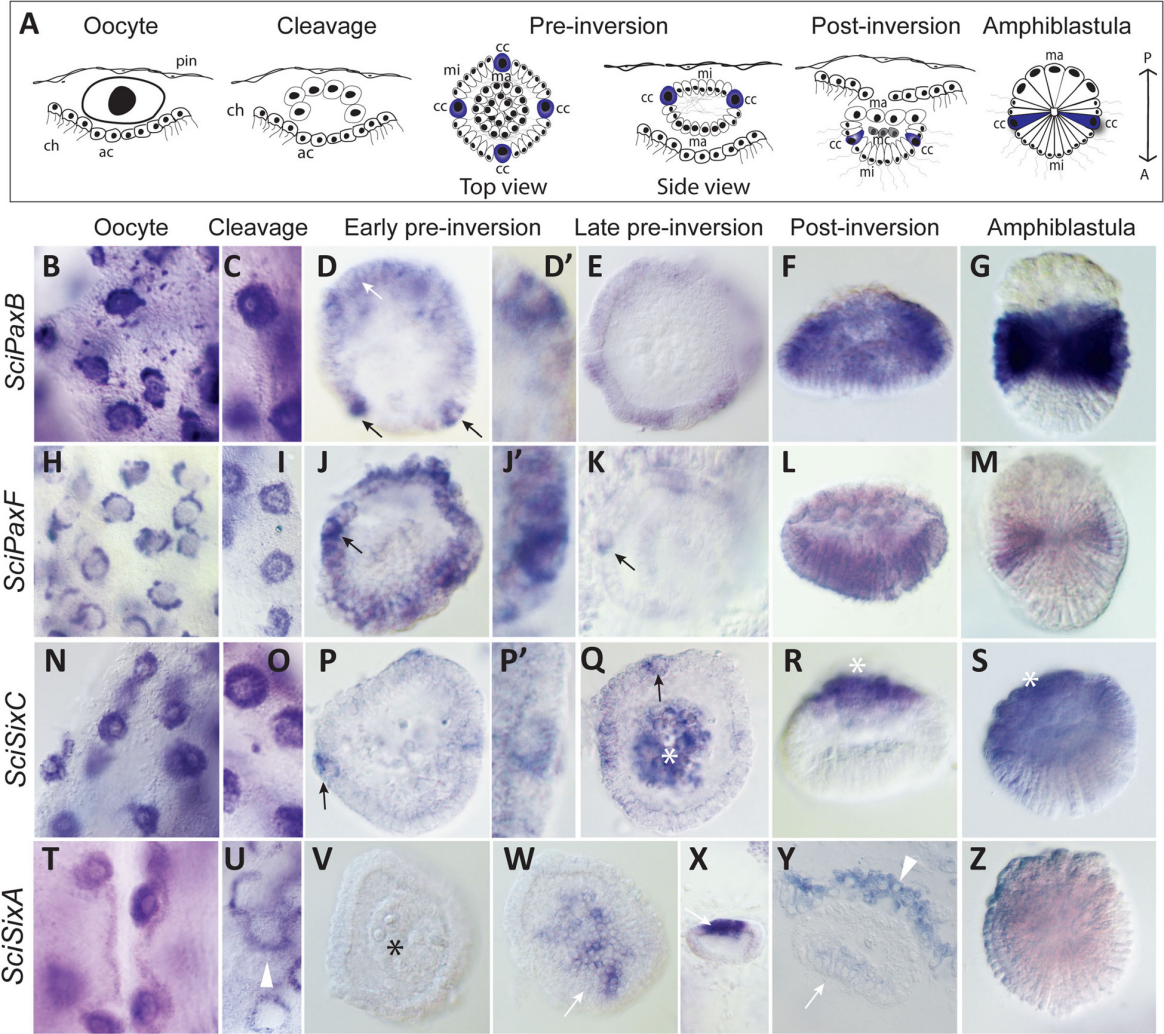
**Expression of *Sycon Pax *and *Six *genes during embryogenesis. A**, Summary of embryogenesis in *Sycon*, with consecutive stages depicted from left to right: oocyte, cleavage, pre-inversion, post-inversion, larva. Abbreviations: pin, pinacocytes; ch, choanocytes; ac, accessory cells; cc, cruciform cells; mi, micromeres ma, macromeres; and mc, maternal cells. **B**-**G**, *SciPaxB* expression is detectable in the oocytes (B), and in all blastomeres during late cleavage (C). During early pre-inversion it is detectable in the micromeres and cruciform cells and in late pre-inversion (E), post-inversion (F) and in larvae (G) *SciPaxB* expression is restricted to the equatorial micromeres. **H**-**M**, *SciPaxF* expression is detectable in the oocytes (H) and is gradually restricted to become predominant in the cross cells (J to K), and then the equatorial micromeres in the larva (M). **N**-**S***, SciSixC* expression is present in the oocytes (N) and then all blastomeres (O), but strongest in the cruciform cells by early pre-inversion (P). During late pre-inversion (Q) the expression is localized to the cruciform cells and the macromeres, and becomes limited to the macromeres in the post-inversion embryos and larva (R,S). **T-Z**, *SciSixA* is expressed in the oocytes (T), but not in cleavage stages or early pre-inversion stage embryos (U, V), when strong expression in a ring of accessory cells is apparent (U). Transient expression in the anterior micromeres is detectable in post-inversion stage embryos (W-Y) but not larvae (Z). All images are of whole mounted, glycerol-cleared specimens, except Y, which is a resin section. Embryos in pre-inversion, post-inversion and larvae were isolated from the tissue, except from K. The asterisk indicates macromeres at the posterior pole of the embryo and larva. Black arrows, white arrows and white arrowheads indicate the cruciform cells, micromeres and the accessory cells, respectively.

As described for multiple species of calcaronean sponges [25,29,51] and schematically represented on Figure 2A, embryogenesis of *Sycon* takes place in the mesohyl. Symmetric cleavage followed by cell differentiation leads to formation of a cup-shaped embryo composed of three cell types: macromeres, micromeres and cross cells. Macromeres are cells which are large, non-ciliated and granular in appearance, located close to the choanocytes of the parent sponge. Numerous micromeres are small and ciliated and located closer to the pinacocytes. The four cruciform cells are similar in size to the macromeres and are symmetrically distributed among the micromeres on the equator of the embryo. The embryo undergoes inversion while it translocates to the radial chamber and the mature larva swims through the oscular opening.

The expression of *SciPaxB* (Figure 2B-G) begins in the oocytes and continues during early and late cleavage, with transcripts present in all blastomeres (Figure 2C). During early pre-inversion, *SciPaxB* expression is evident in the cruciform cells and micromeres (Figure 2D, D’). In the larvae, *SciPaxB* expression is high in a broad band of equatorial micromeres (Figure 2F). The expression of *SciPaxF* begins in the oocytes (Figure 2H) and is also initially uniform in all blastomeres, but by early pre-inversion the expression is stronger in micromeres and evident in the cruciform cells (Figure 2J-K). In the larvae, the *SciPaxF* expression is weak in the posterior micromeres, and expression in the cruciform cells is no longer apparent (Figure 2M). Expression of *SciSixC* is detected in the oocytes (Figure 2N), and during early pre-inversion it becomes most prominent in the cruciform cells (Figure 2P), resolving to strong expression in the macromeres and weaker in the cruciform cells during late pre-inversion (Figure 2Q). In post-inversion stages and larva, only macromere expression is apparent (Figure 2R, S).

The expression of *SciSixA* is strong in oocytes (Figure 2T), but not detectable in the cleavage stage embryos (Figure 2U-V), instead becoming prominent in a ring of accessory cells (which are derived from choanocytes) surrounding the embryos (Figure 2U, Y). In the late pre-inversion stage embryos, *SciSixA* expression is detectable in the anterior micromeres of the embryos (Figure 2W-Y) although this expression is no longer detectable in the larvae (Figure 2Z). Finally, the *SciSixB* gene expression was weak and appeared ubiquitous throughout all cell types with no distinct expression pattern (data not shown). No expression of *SciEya* was observed during embryogenesis, although its expression is detectable in choanocytes (Additional file 10 and see below).

### Pax-Six-Eya are co-expressed in choanocyte chambers

**Figure 3:**
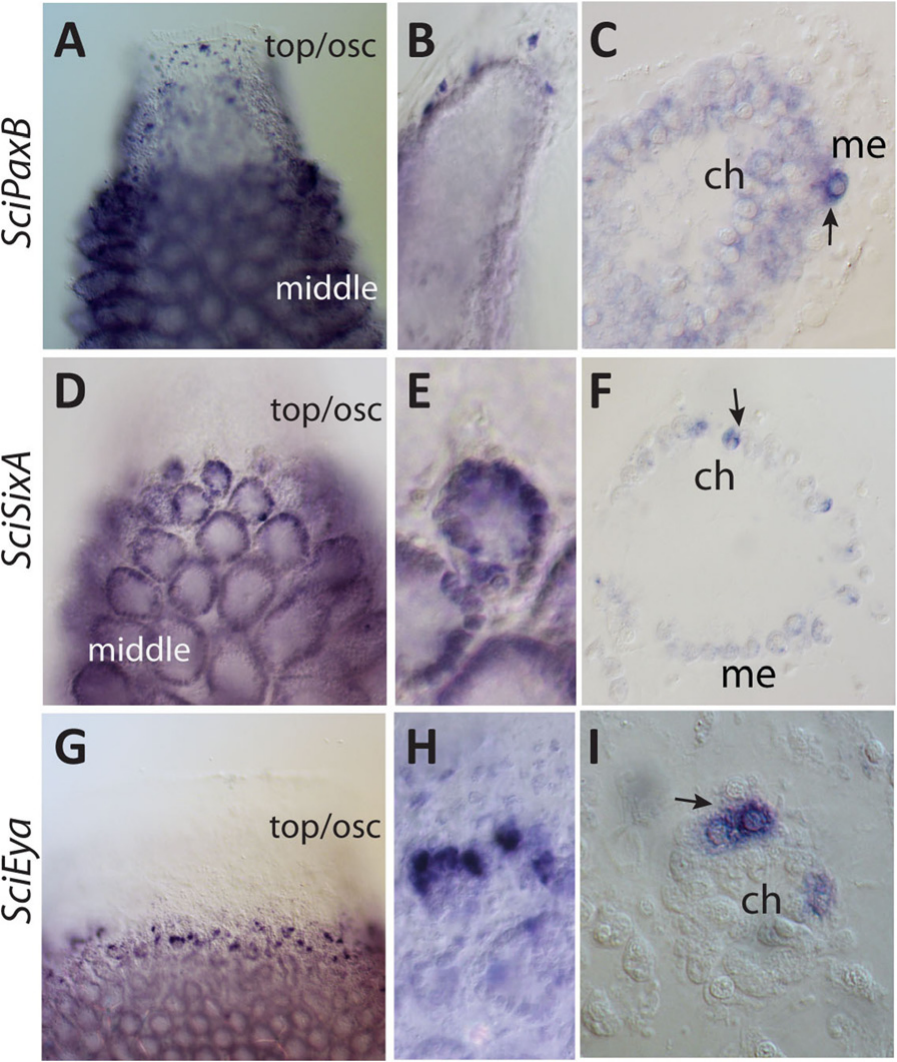
**Expression of *Pax, Six *and *Eya *genes in adult sponges. A**-**C**; *SciPaxB* is expressed in all choanocytes and a fraction of mesohyle cells. **D**-**F**, *SciSixA* and **G**-**I**, *SciEya*, are expressed in choanocytes of the radial chambers, but not choanocytes located in the upper region remaining in asconoid organization. A, D and G are upper parts of whole young sponges, B, E and H are magnifications of radial chambers, C, F and I are plastic sections through the radial chambers. Abbreviations: me, mesohyl cells; ch, choanocytes; arrows indicate strongest expression.

In addition to the embryonic expression described above, *SciPaxB, SciSixA* and *SciEya* transcripts are detected in adult cell types, being most prominent in the choanocytes (the innermost epithelial cells responsible for feeding) (Figure 3). Expression of *SciPaxB* is uniform throughout the choanocytes and is strong in scattered mesohyl cells, particularly at the tips of the radial chambers and in the area of osculum (Figure 3A-C). *SciSixA* expression is stronger in choanocytes of the uppermost, forming chambers than in the older chambers, with somewhat heterogeneous expression among the choanocytes (Figure 3D-F). Notably, *SciSixA* expression is not detectable in choanocytes which are not organized in chambers, but located in the uppermost area of the sponge in the region of asconoid organization (Figure 3D). The expression of *SciEya* is similar to that of *SciSixA* in that the choanocytes of the oscular region are negative, the choanocytes located in the uppermost, forming choanocyte chambers display the most prominent expression, and the choanocytes of the older chambers display low level of expression (Figure 3G-I).

### Elav and Musashi are expressed in the cruciform cells and macromeres

**Figure 4:**
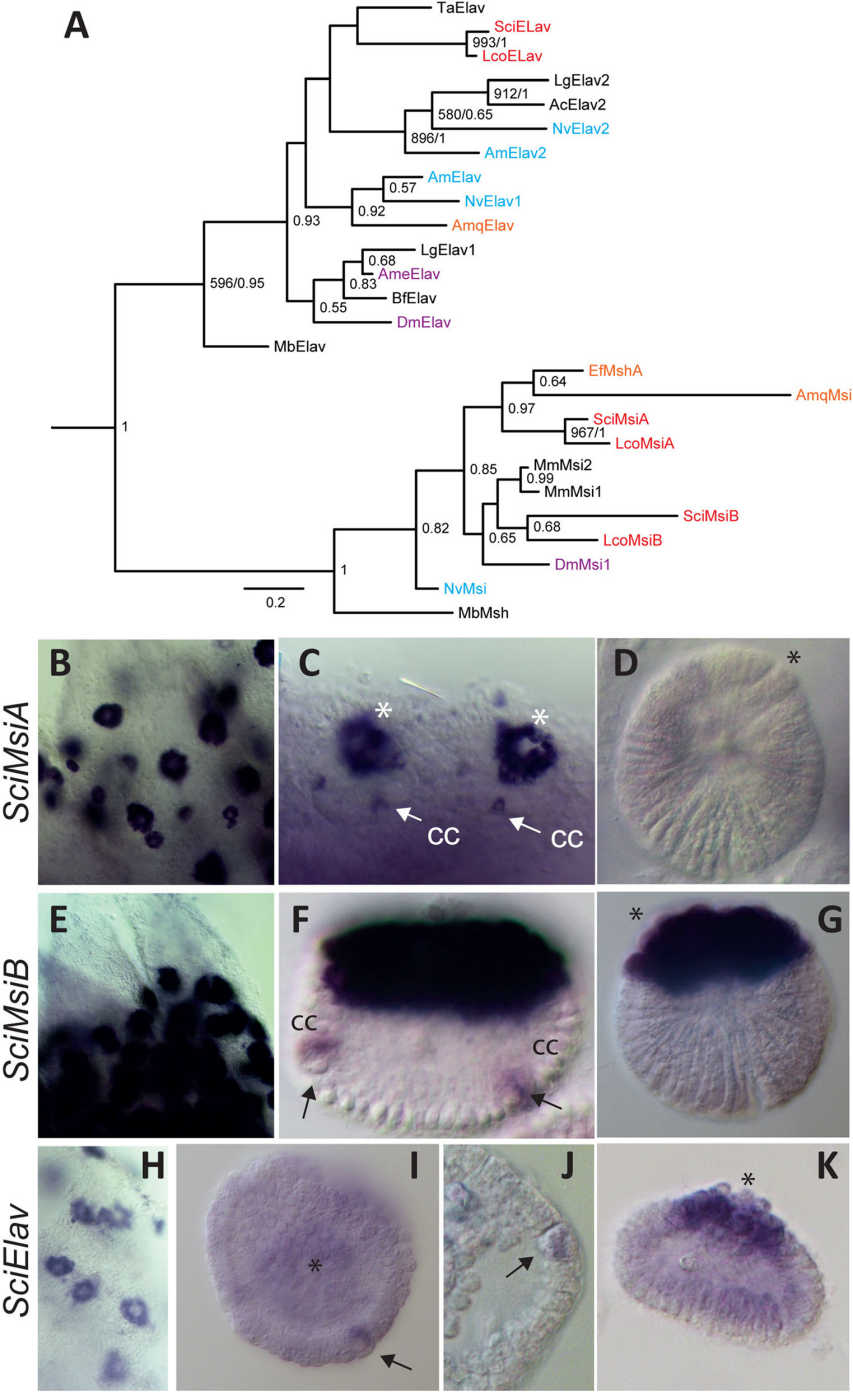
**Phylogenetic tree and embryonic expression of *S. ciliatum Msi *and *Elav *genes. A**, This is a Bayesian tree inferred from the RRM2 motif of the RNAbp, *Elav* and *Musashi*. ML bootstrap values greater than 500 and posterior probabilities generated by MrBayes greater than 0.5 are displayed. The tree was rooted with the Pabp subfamily. Names are prefixed as in Figure 1 except from: Ac, *Aplysia californica*; Ame, *Apis mellifera*; Mb, *Monosiga brevicollis*; Lg, *Lottia gigantea* and Ta, *Trichoplax adhaerens*. **B**–**K** Whole mount *in situ* hybridization: B-D, *SciMsiA* expression in sponges containing oocytes (B); embryos during pre-inversion showing expression in the cruciform cells (cc, arrows) and macromeres (C) and no expression in the larva (D). E-G, *SciMsiB* expression in oocytes (E), in cc and macromeres in embryos during post-inversion (F), and expression in the macromeres in the larva (G). H-K, *SciElav* whole mount *in situ* of sponges containing oocytes (H); during pre-inversion *SciElav* is seen in cruciform cells and weak in macromeres (I, J); and by post-inversion *SciElav* expression is predominant in macromeres (K). The asterisk indicates macromeres at the posterior pole of the embryo and larva.

We identified one *Elav* and two *Musashi* genes in each of the two analyzed sponges (Additional file 11). The phylogenetic analyses were performed using the conserved RRM2 domain present in both gene families (Additional file 12). The calcisponge *Elav* genes affiliated with the *Trichoplax adhaerens Elav* gene, albeit with poor support. Affiliation of this clade with either *Elav1* or *Elav2* subfamily differed between ML and Bayesian analysis, never reaching significant support (Figure 4A). The calcisponge *MsiA* sequences clustered with demosponge *Msi* genes, while the *MsiB* pair affiliated with bilaterian sequences (Figure 4A).

Both of *Msi* genes and the *Elav* gene display similar expression patterns, being expressed strongly and uniformly in the oocytes and during cleavage, and gradually becoming most prominent in the cruciform cells (Figure 4B, E, H, I and Additional file 13). During pre-inversion, strong expression of *SciMsiA* and weaker expression of both *SciMsiB* and *SciElav* is also detectable in the macromeres, in addition to continued expression in the cruciform cells (Additional file 13 and Figure 4C, J). Among the three genes, *SciMsiB* is detectable until the latest (post-inversion) stages in the cruciform cells (Figure 4F). Finally, *SciMsiB* and *SciElav* are detectable in the larval macromeres (Figure 4G, K).

## Discussion

*Pax*, *Six*, *Eyes absent* and *Dachshund* genes are involved in a variety of developmental processes, including the determination and morphogenesis of eyes and other sensory organs such as the ear [3,52]. In addition to overlapping expression patterns, protein-protein interactions and direct expression regulation demonstrate that PSED genes function as a network in a variety of animals [1,3]. Although many of the regulatory relationships are conserved in the PSED network, the interactions between network components can vary during development and in different animal lineages [6,53].

**Figure 5:**
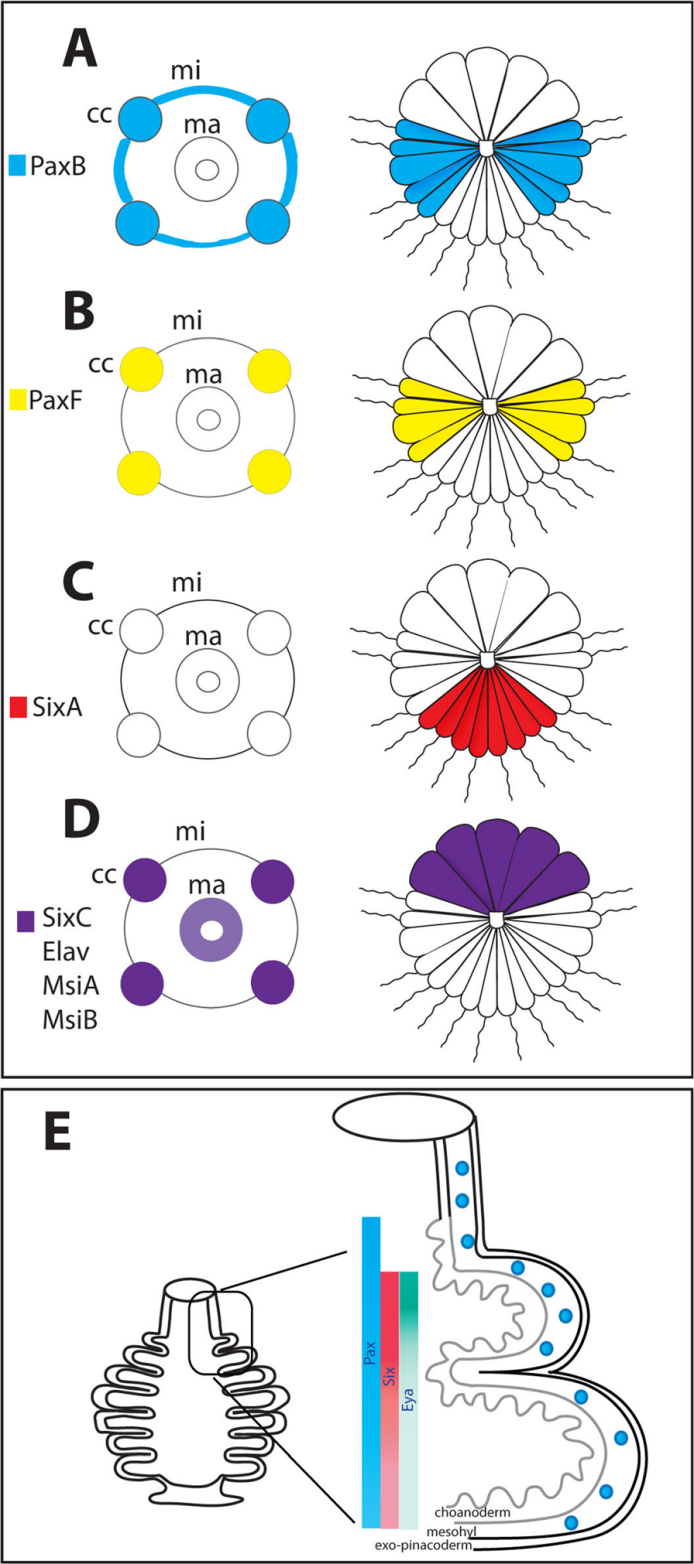
**Summary of the expression patterns. A**, *SciPaxB* in cruciform cells and equatorial micromeres during pre-inversion and micromeres in the larvae; **B**, *SciPaxF* in cruciform cells during pre-inversion and equatorial micromeres of the larva; **C**, *SixA* in anterior micromeres, **D**, *SciSixC*, *SciElav*, *SciMsiA* and *SciMsiB* in the cruciform cells and in the macromeres of the post-inversion stage embryos and/or larvae; **E**, Gradients of *SciSixA* and *SciEya* in choanocyte cells organized in chambers, *SciPaxB* in all choanocytes and in scattered mesohyl cells.

The origin of the PSED network has been previously investigated in non-bilaterian animals, including cnidarians [7-9,13,54] and demosponges [16,47]. As demosponges contain *PaxB* and *Six1/2* genes, but not *Eya* or *Dach*, it has been proposed that the ancestral network was composed of these two genes only. However, absence of *Eya* in demosponges appears secondary, as it is present in the choanoflagellates [13]. Here we show that the genomes of calcisponges also contain *Eya*, suggesting that its loss occurred in the lineage leading to demosponges and is thus not representative of all sponges. A question remains whether the three genes (*Pax-Six-Eya*) indeed do interact, and thus form a network, or whether the network arose later during evolution from components working individually in sponges. Importantly, a recent study in the demosponge *Ephydatia mulleri* indicated that in this species expression of *Six1/2* might be controlled by *PaxB*[17]. Unfortunately, it is not possible at the moment to functionally test interactions of the potential network components in calcisponges. However, their co-expression in several developmental contexts in *Sycon* suggests that these genes might also operate as a network, and in fact might control similar developmental processes as in other animals: sensory cell specification and morphogenesis. With the exception of *Eya*, all of the identified genes are co-expressed in the oocytes and in embryos undergoing cleavage, suggesting they are maternally expressed and have early developmental roles. In subsequent stages of development, the most striking and potentially informative co-expression is in the embryonic cruciform cells and later during adult morphogenesis, as summarized on Figure 5.

The cruciform cells have been suggested to act as larval photoreceptors [33] and the amphiblastulae have been shown to respond to light [55]. Surprisingly, we found that not only opsin, but also cryptochrome genes, which likely convey light sensitivity to the demosponge larvae, are absent from *Sycon* and *Leucosolenia* genomes, indicating that calcisponges have lost cryptochrome. Thus, calcisponge larvae might be able to detect light using another, yet unclear mechanism. Intriguingly, homologues of multiple genes involved in specification of neural and sensory cells in other animals are expressed during cross cell differentiation. These include *SciPaxB, SciPaxF*, *SciSixC*, *SciEla*v, *SciMsiA* and *SciMsiB* presented in this study (see Figure 5A), *SciSoxB*[24], components of the Wnt pathway (*Dvl*, *Tcf* and *Beta-catenin*), *Smad1/5* and *Nanos*[25].

The observed co-expression indicates that *SciSixC*, *SciPaxB* and *SciPaxF* may potentially interact during embryogenesis, while *SciPaxB, SciSixA and SciEya* may potentially interact during adult morphogenesis (Figure 5). Among the three potential components of the PSEN in *Sycon* expressed in the choanocytes, *SciPaxB* has the broadest expression, with transcripts uniformly detected in all choanocytes. In contrast, *SciSixA* and *SciEya* transcripts are conspicuously absent from the uppermost choanocytes remaining in asconoid organization, but are particularly strongly expressed in the choanocytes of the uppermost chambers which are undergoing morphogenesis, with *SciEya* expression diminishing along a somewhat steeper gradient than *SciSixA*, so that both are expressed at low levels in the already formed chambers (Figure 5b). *SciPaxB*, *SciSixA* and *SciEya* expression patterns are thus consistent with interaction of these three genes during formation and maintenance of the organization of the radial chambers, and thus with a concerted role in morphogenesis. Despite lack of *Eya* of the demosponge *Ephydatia*, the morphogenetic role of the potential network might be a conserved feature, as knock down of *EmPaxB* and *EmSix1/2* results in apparent dysmorphogenesis of the juveniles [17].

## Conclusions

Overall, the presence of the sponge *Eya* gene and co-expression of *Pax*, *Six* and *Eya* genes in calcisponges indicate that the *Pax-Six-Eya* network may have already been established in the last common ancestor of sponges and eumetazoans, with *Eya* subsequently lost in demosponges. Based on gene expression during adult body plan formation in *Sycon*, we propose that this network had an ancient role in morphogenesis. Additionally, co-expression of *Pax* and *Six* with conserved eumetazoan neural genes *Elav* and *Msi* in candidate larval sensory cells, suggests these genes could be ancestrally involved in the determination of sensory cell types. We envisage a scenario in which a simple PSE network was active in early metazoans, and that additional genes, such as *Dachshund*, were then later co-opted into the network to expand its regulatory capacity in more complex animals.

## Abbreviations

BLAST: Basic Local Alignment Search Tool; ED: *Eya* domain; ML: Maximum Likelihood; PD: Paired domain; PSEDN: *Pax-Six-Eya-Dach* network; RDGN: Retinal Determination Gene Network.

## Competing interests

The authors declare that they have no competing interests.

## Authors’ contributions

SF and MA conceived and designed the study. MA and SL carried out field collection. SF performed phylogenetic analyses and analyzed expression of all genes presented in this manuscript except *Musashi*, which were studied by SL. SF drafted and MA edited the manuscript with input from SL. All authors read and approved the final manuscript.

## Supplementary Material

Additional file 1 **The predicted Pax protein sequences and intron-exon boundaries.** Exon-intron boundaries are highlighted in yellow, the paired domain is red. Click here for file

Additional file 2 **Alignment of the PD domain.** This alignment was used for the phylogenetic analyses displayed in Figure 1C. The red box indicates the location of the RED motif used for the phylogenetic analyses in Additional file 3. Abbreviations are as in Figure 1. Click here for file

Additional file 3 **Bayesian phylogenetic tree of the *Pax* gene family inferred from the RED motif of the PRD domain.** Support values on nodes are as follows: left, bootstrap (BT) values obtained from ML analysis; right, posterior probability from the Bayesian analysis. For abbreviations of species names see Figure 1. Click here for file

Additional file 4 ***Six *** **genes protein sequences in *Sycon* and *Leucosolenia.*** Exon-intron boundaries are indicated by highlighting. Sine oculis domain is red, the homeodomain is underlined. Click here for file

Additional file 5 **Alignment of the homeodomain of the SINE and TALE classes including all of Six genes and selected TALE genes identified in calcisponges.** Abbreviations are as in Figure 1. Click here for file

Additional file 6 **Maximum likelihood tree of the SINE class.** Phylogenetic tree inferred from the homeodomain of Six and TALE genes. Bootstrap values are displayed on each node. Names are prefixed as in Figure 1. The tree was rooted with a selection of TALE class of homeobox genes. *Mnemiopsis* Six genes found in long branches on the tree from Figure 1B were not included in this analysis. Click here for file

Additional file 7 **Maximum likelihood phylogenetic analyses of sine oculis domain and homeodomain of the Six class.** ML bootstrap values greater than 500 are displayed. Names are prefixed as in Figure 1. Click here for file

Additional file 8 **EYA protein sequences.** Red indicates the location of the ED domain. Exon-intron boundaries are highlighted. Click here for file

Additional file 9 **Alignment of the ED domain.** This alignment was used for the phylogenetic analyses displayed in Figure 1B. Abbreviations are as in Figure 1. Click here for file

Additional file 10 **The *SciEya *** **gene is not expressed during embryogenesis.** A, oocytes; B, embryos during pre-inversion and C, post-inversion. Click here for file

Additional file 11 **Elav and Msi protein sequences.** Exon-intron boundaries are highlighted. Red indicates the location of the RMM2 domain. Click here for file

Additional file 12 **RRM2 motif alignment for Msi and Elav sequences.** This alignment, without gaps, was used for the phylogenetic analyses displayed in Figure 4. Click here for file

Additional file 13 **Predominant expression of *SciMsiA* and *SciElav* in the cruciform cells.** Late cleavage and pre-inversion stage embryos are shown for *SciMsiA* and *SciElav*, respectively. Click here for file
