# Supplementary figures and images for: Evolution of the Pax-Six-Eya-Dach network: the calcisponge case study
Source: EvoDevo. 2014 Jun 23;5:23. doi: 10.1186/2041-9139-5-23 (PMC4083861; doi:10.1186/2041-9139-5-23)

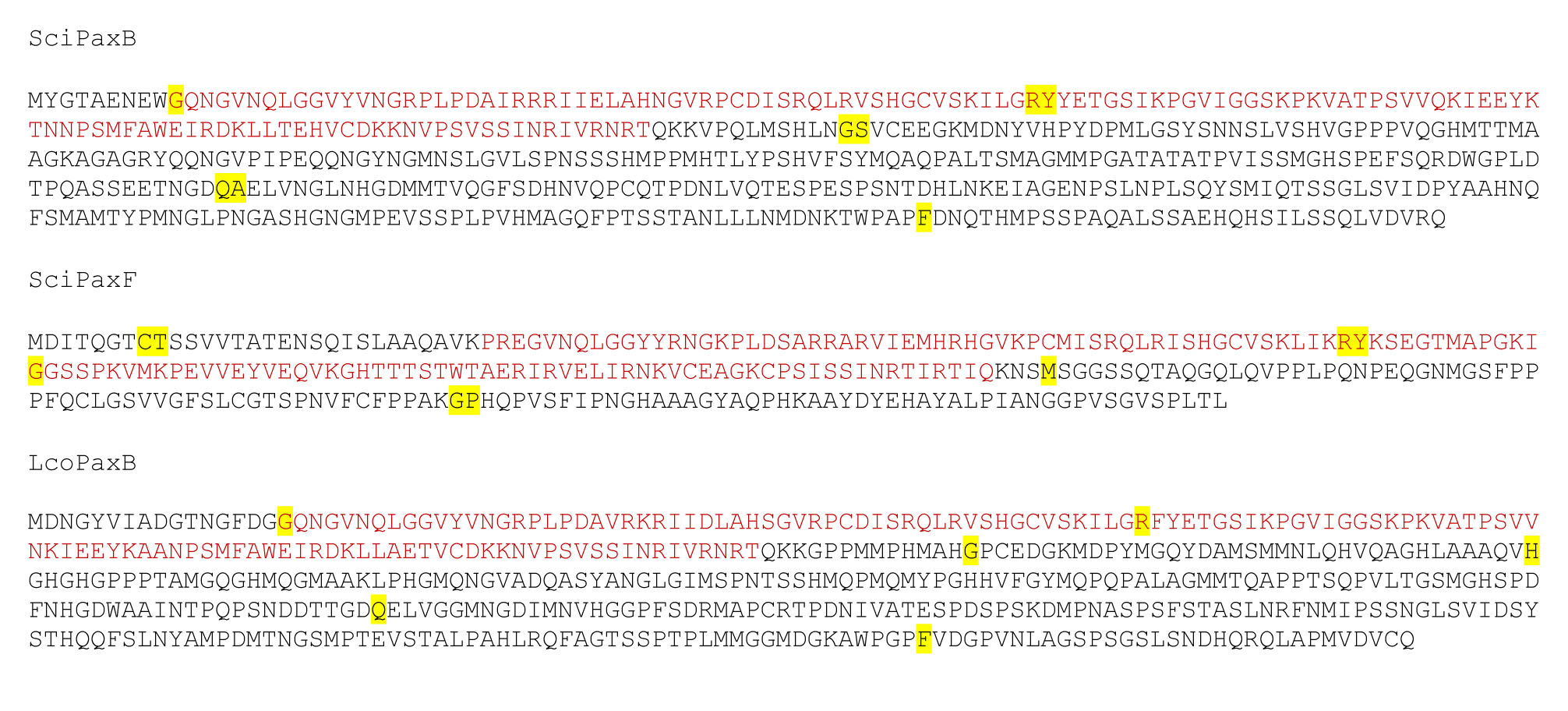

Supplement: Additional file 1 — The predicted Pax protein sequences and intron-exon boundaries. Exon-intron boundaries are highlighted in yellow, the paired domain is red. [file 2041-9139-5-23-S1.jpeg]

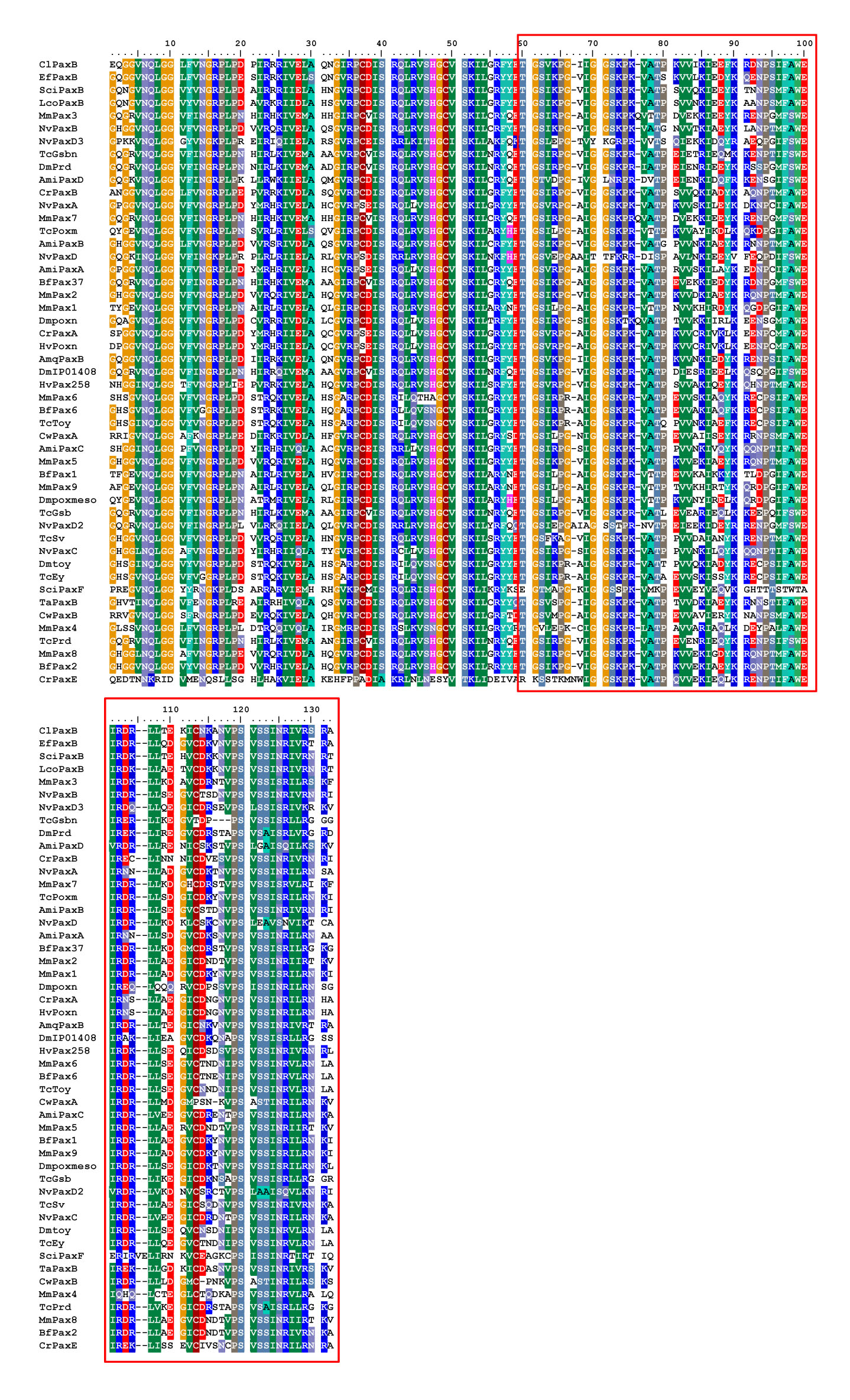

Supplement: Additional file 2 — Alignment of the PD domain. This alignment was used for the phylogenetic analyses displayed in Figure 1C. The red box indicates the location of the RED motif used for the phylogenetic analyses in Additional file 3. Abbreviations are as in Figure 1. [file 2041-9139-5-23-S2.jpeg]

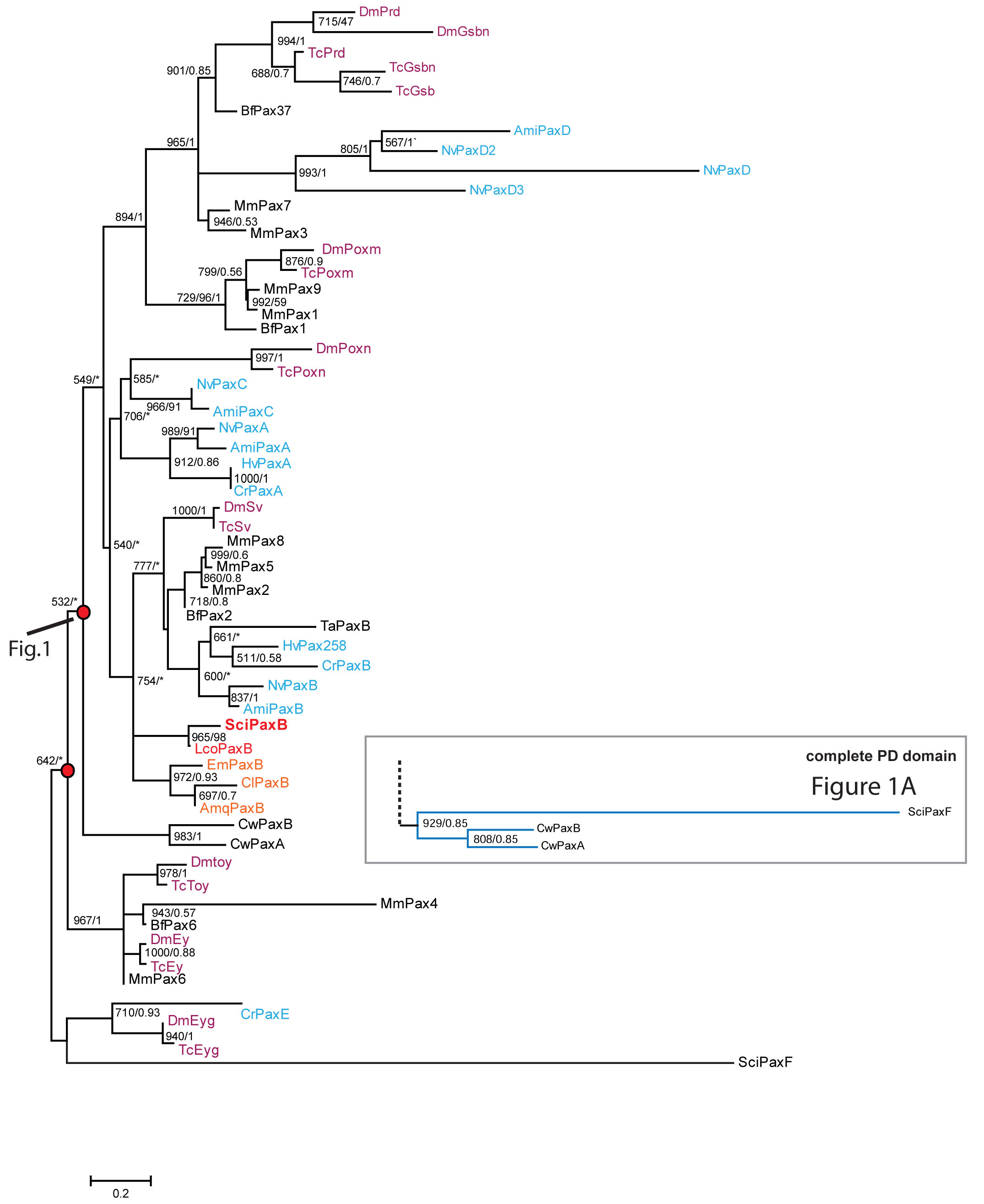

Supplement: Additional file 3 — Bayesian phylogenetic tree of the Pax gene family inferred from the RED motif of the PRD domain. Support values on nodes are as follows: left, bootstrap (BT) values obtained from ML analysis; right, posterior probability from the Bayesian analysis. For abbreviations of species names see Figure 1. [file 2041-9139-5-23-S3.jpeg]

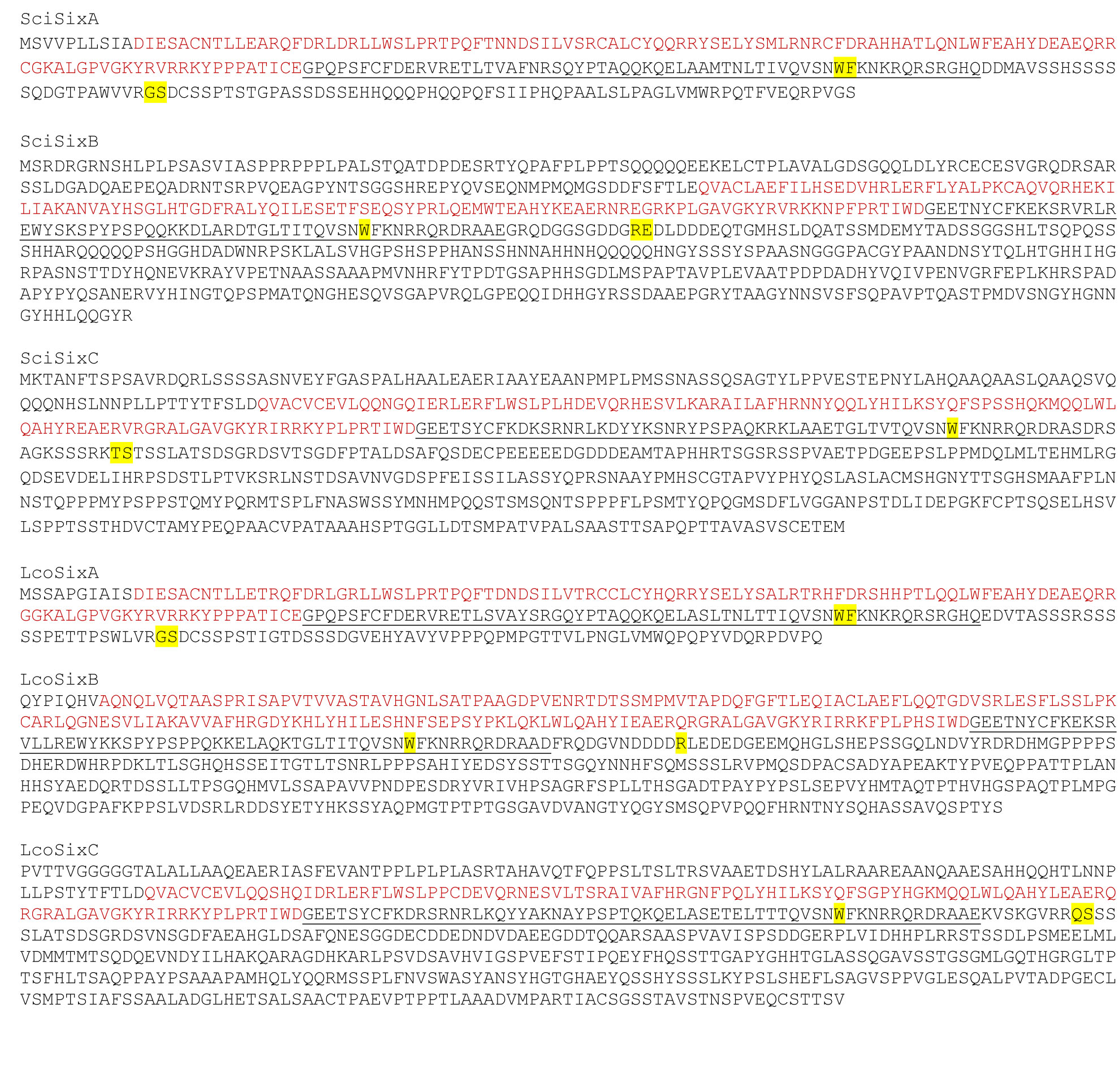

Supplement: Additional file 4 — Six genes protein sequences in Sycon and Leucosolenia. Exon-intron boundaries are indicated by highlighting. Sine oculis domain is red, the homeodomain is underlined. [file 2041-9139-5-23-S4.jpeg]

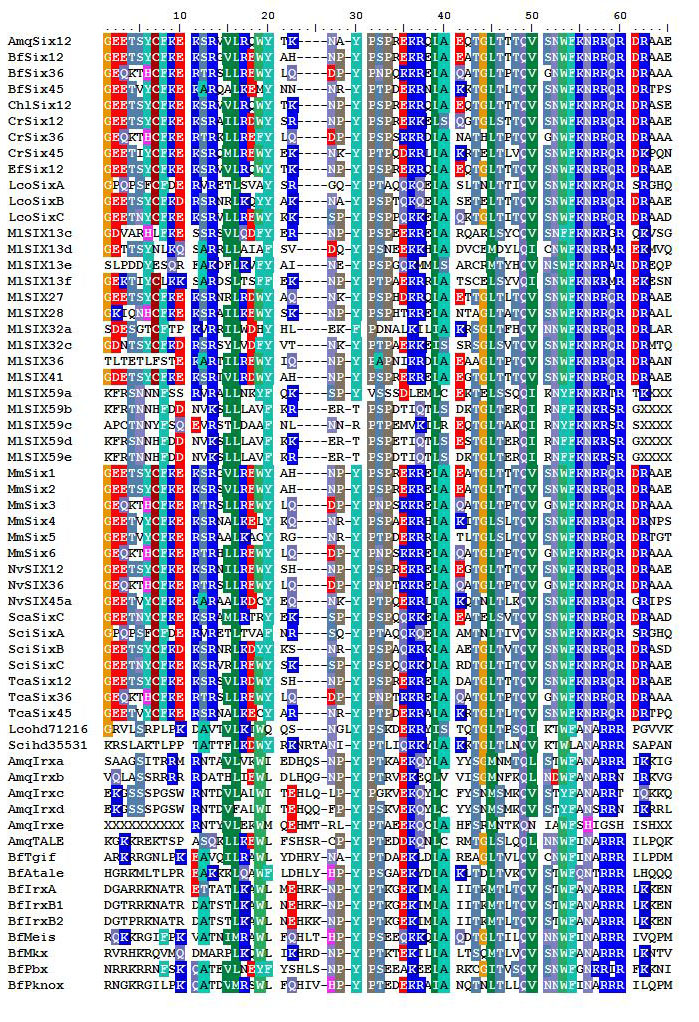

Supplement: Additional file 5 — Alignment of the homeodomain of the SINE and TALE classes including all of Six genes and selected TALE genes identified in calcisponges. Abbreviations are as in Figure 1. [file 2041-9139-5-23-S5.jpeg]

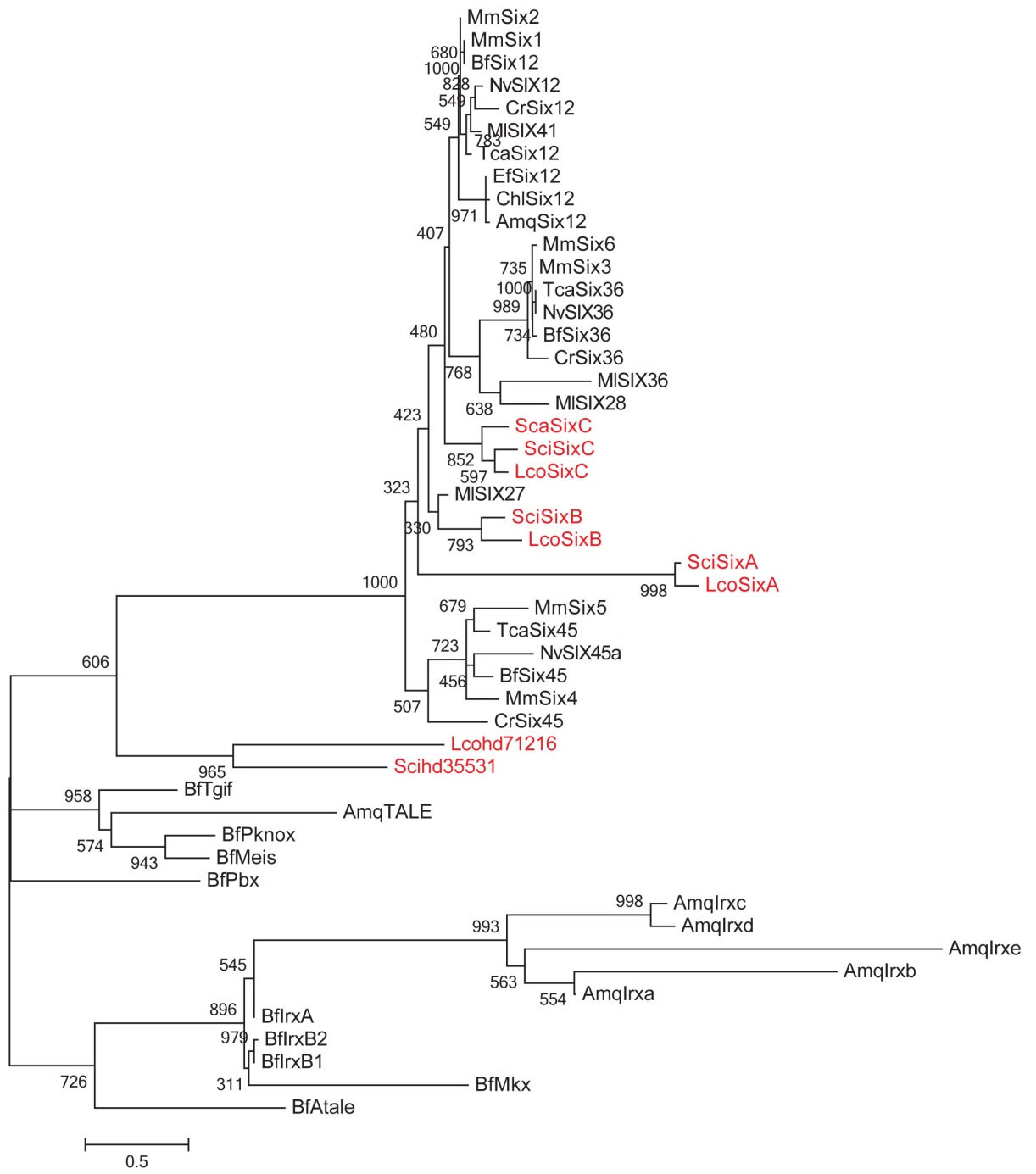

Supplement: Additional file 6 — Maximum likelihood tree of the SINE class. Phylogenetic tree inferred from the homeodomain of Six and TALE genes. Bootstrap values are displayed on each node. Names are prefixed as in Figure 1. The tree was rooted with a selection of TALE class of homeobox genes. Mnemiopsis Six genes found in long branches on the tree from Figure 1B were not included in this analysis. [file 2041-9139-5-23-S6.jpeg]

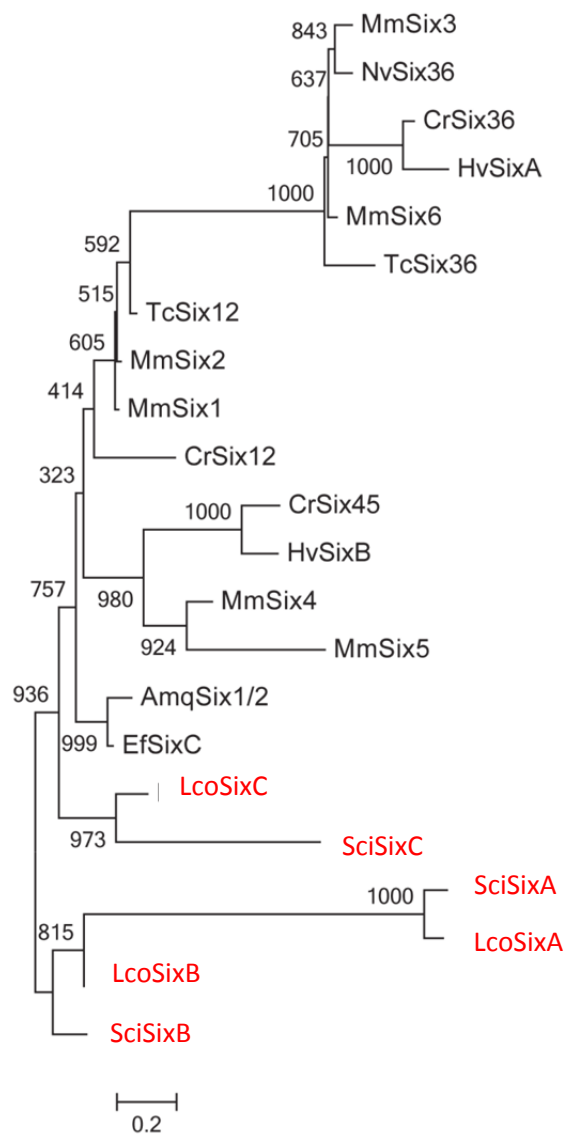

Supplement: Additional file 7 — Maximum likelihood phylogenetic analyses of sine oculis domain and homeodomain of the Six class. ML bootstrap values greater than 500 are displayed. Names are prefixed as in Figure 1. [file 2041-9139-5-23-S7.pdf]

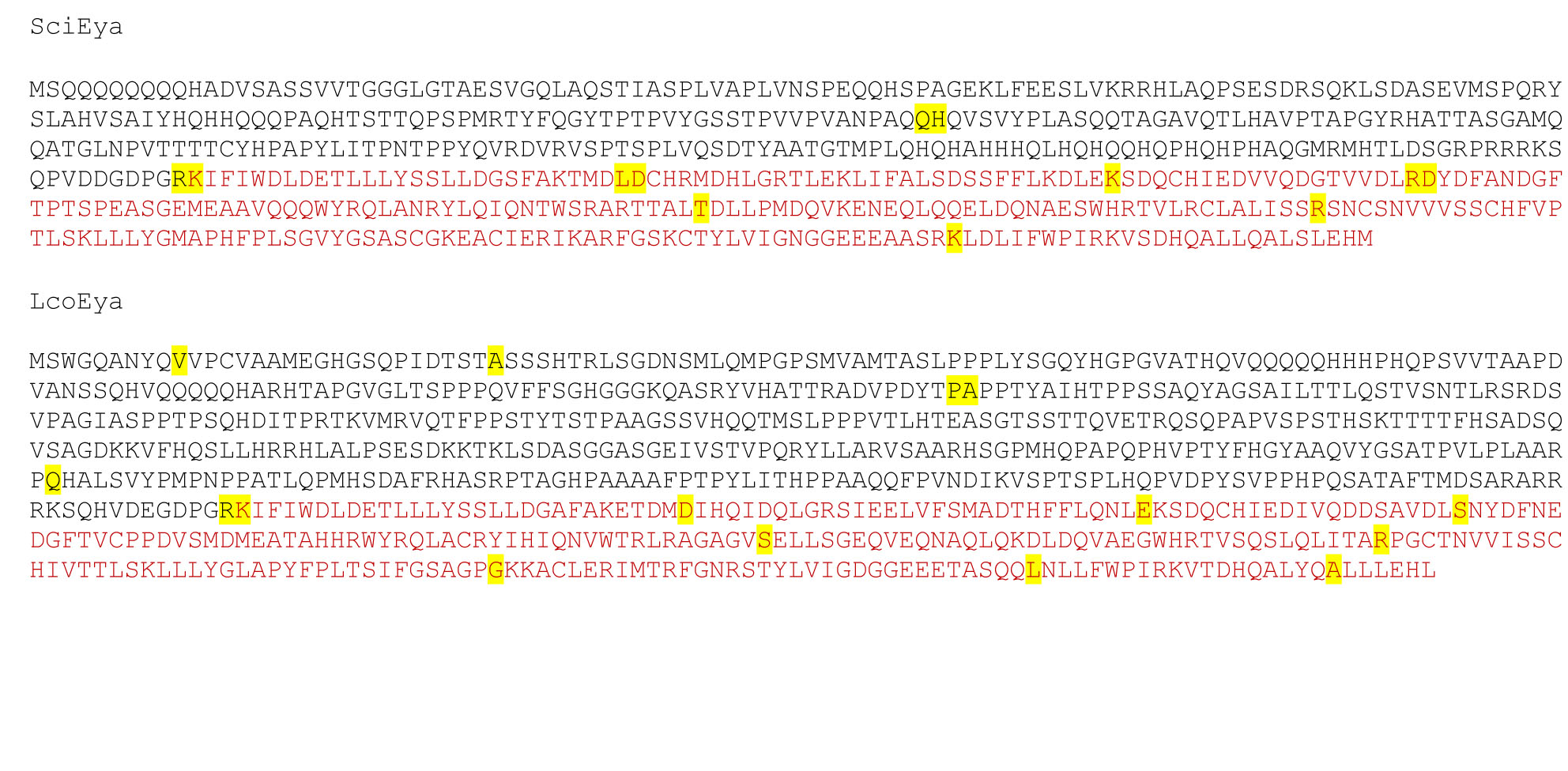

Supplement: Additional file 8 — EYA protein sequences. Red indicates the location of the ED domain. Exon-intron boundaries are highlighted. [file 2041-9139-5-23-S8.jpeg]

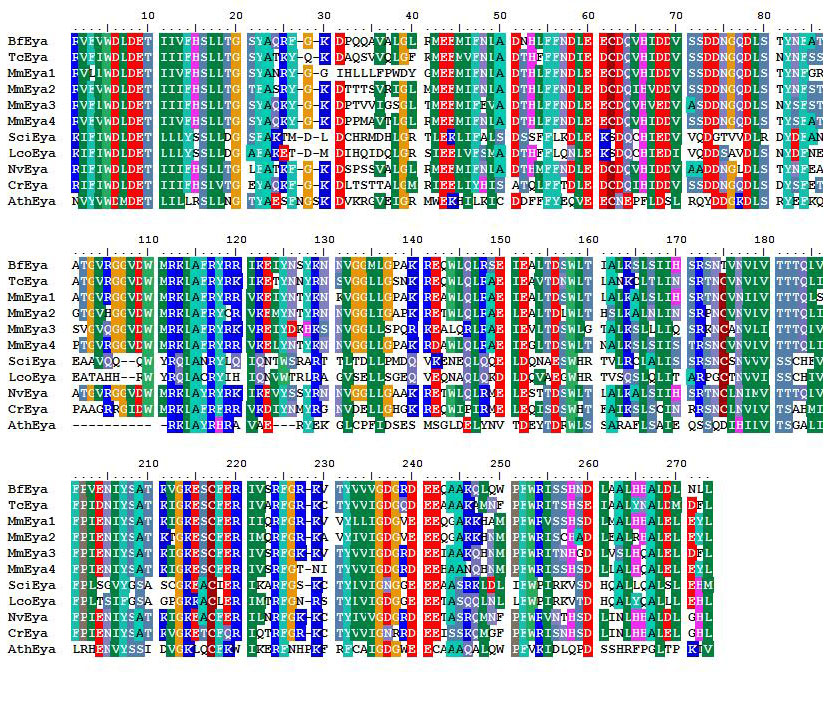

Supplement: Additional file 9 — Alignment of the ED domain. This alignment was used for the phylogenetic analyses displayed in Figure 1B. Abbreviations are as in Figure 1. [file 2041-9139-5-23-S9.jpeg]

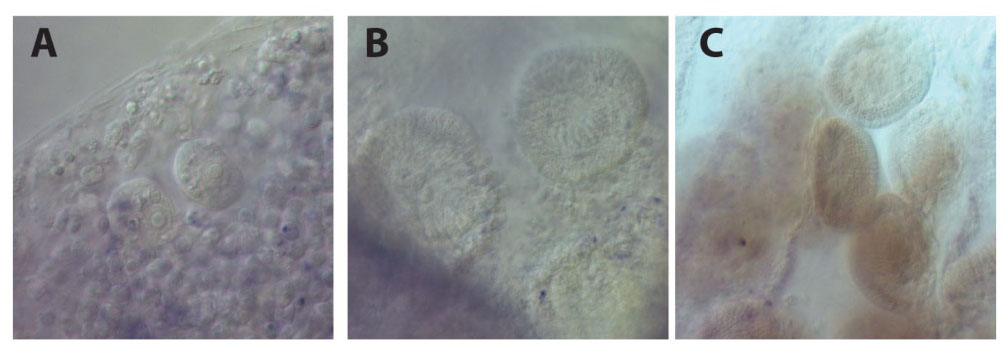

Supplement: Additional file 10 — The SciEya gene is not expressed during embryogenesis. A, oocytes; B, embryos during pre-inversion and C, post-inversion. [file 2041-9139-5-23-S10.jpeg]

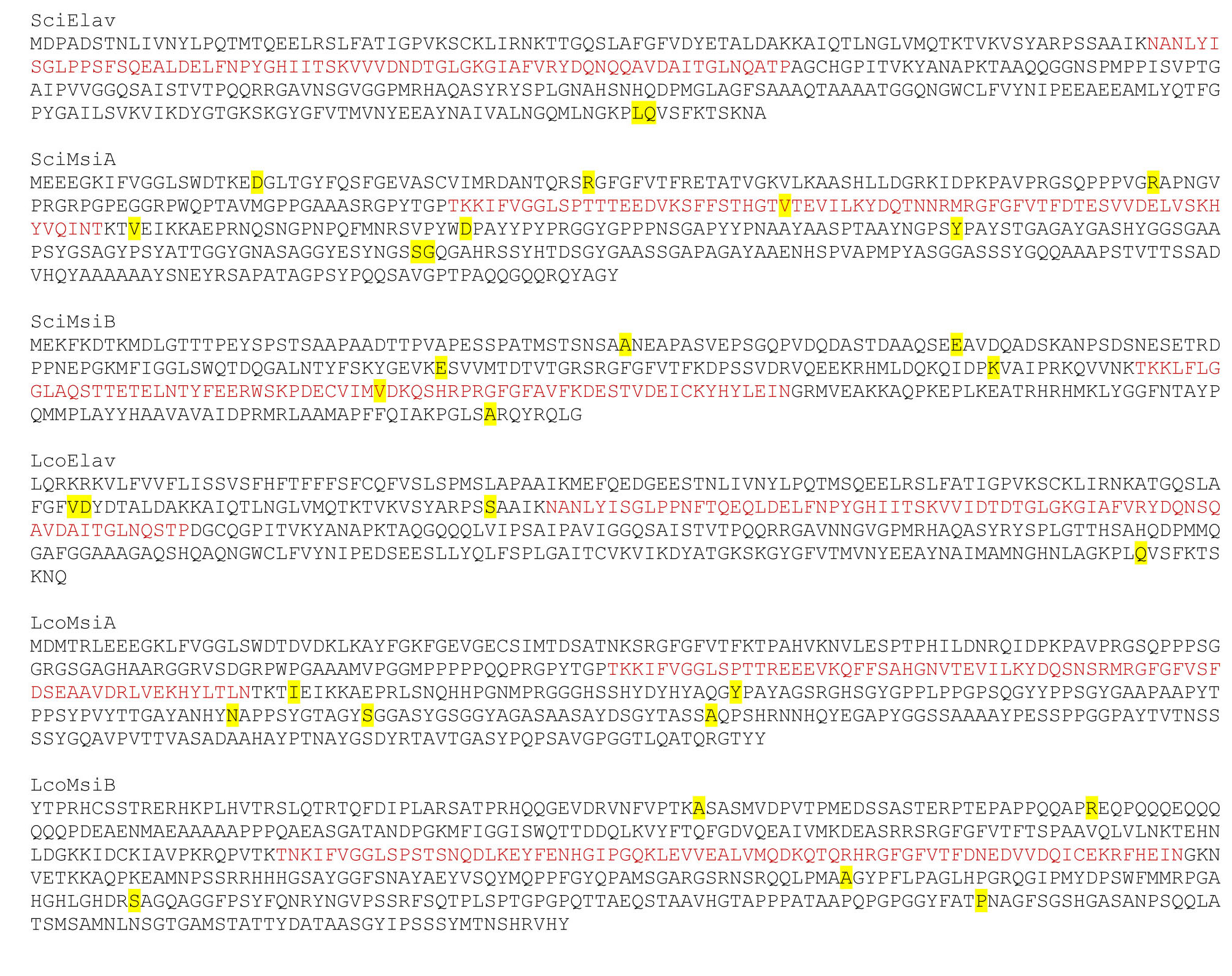

Supplement: Additional file 11 — Elav and Msi protein sequences. Exon-intron boundaries are highlighted. Red indicates the location of the RMM2 domain. [file 2041-9139-5-23-S11.jpeg]

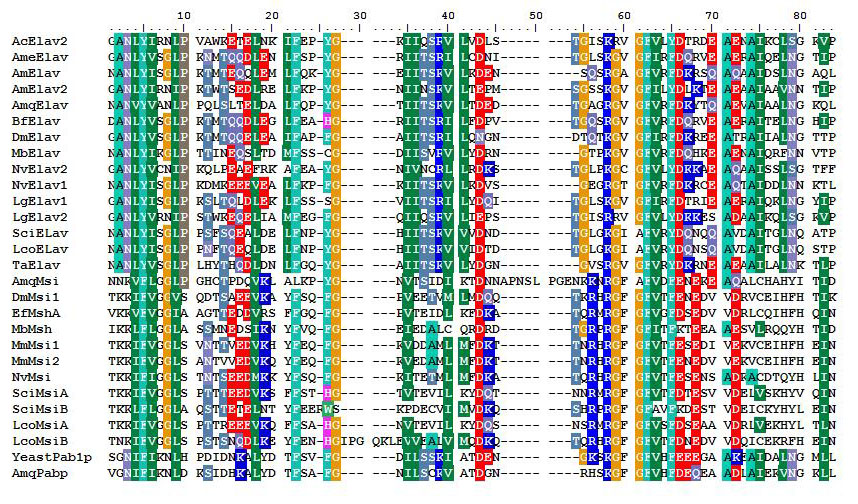

Supplement: Additional file 12 — RRM2 motif alignment for Msi and Elav sequences. This alignment, without gaps, was used for the phylogenetic analyses displayed in Figure 4. [file 2041-9139-5-23-S12.jpeg]

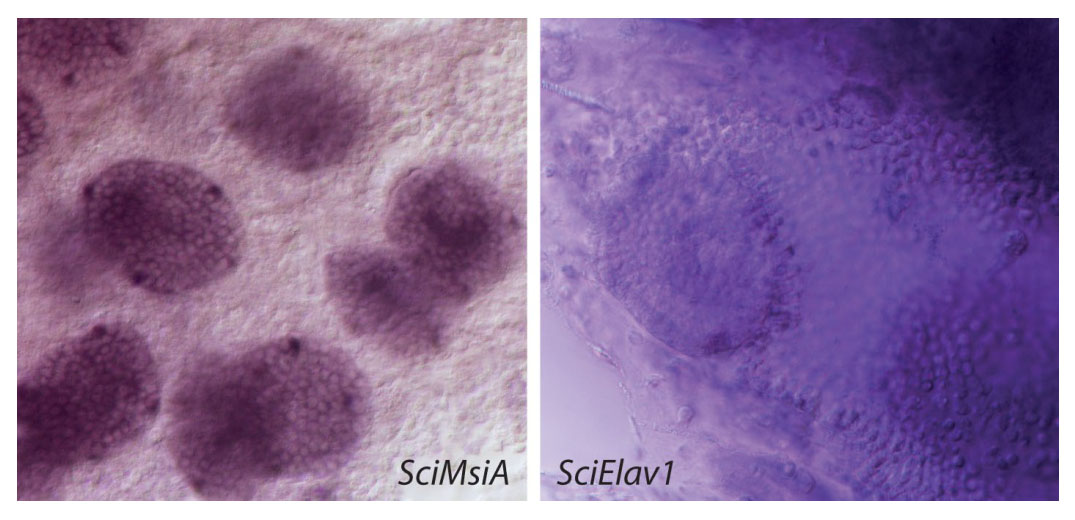

Supplement: Additional file 13 — Predominant expression of SciMsiA and SciElav in the cruciform cells. Late cleavage and pre-inversion stage embryos are shown for SciMsiA and SciElav, respectively. [file 2041-9139-5-23-S13.jpeg]
